# Between their own and the western: the experience of Emberá communities in Chocó with Malaria

**DOI:** 10.17843/rpmesp.2025.424.14846

**Published:** 2025-11-24

**Authors:** Camilo Duque-Ortiz, Stefany Osorno-Sánchez, Suatny Dayana Gutierrez-Asprilla, Maria Jose Garcia-Tirado, Juliana Nieto-Betancurt, Luisa Fernanda Guzman-Sánchez, Sara Montoya-Cedula, Marjorie Pérez-Villa, Luz Elena Botero-Palacio, Cristian Vera-Marin, José Mauricio Hernández-Sarmiento

**Affiliations:** 1 Universidad Pontificia Bolivariana, Medellín, Colombia. Universidad Pontificia Bolivariana Universidad Pontificia Bolivariana Medellín Colombia; 2 Grupo de Investigación Cuerpos y Contextos, Laboratorio De Osteología Antropológica y Forense (LOAF) del Departamento de Antropología, Universidad de Antioquia, Medellín, Colombia. Universidad de Antioquia Grupo de Investigación Cuerpos y Contextos Laboratorio De Osteología Antropológica y Forense (LOAF) del Departamento de Antropología Universidad de Antioquia Medellín Colombia

**Keywords:** Malaria, Mosquito Vectors, Indigenous Population, Traditional Medicine, Social Behavior

## Abstract

**Objective.:**

To explore how the Emberá communities of Chocó, Colombia, coexist with malaria, through the recognition of the meanings they construct about the disease, its treatment, and care.

**Materials and methods.:**

This was an ethnographic study. Between October 2022 and November 2023, 250 hours of participant observation were conducted in six Emberá indigenous communities, and 25 interviews were conducted with community members, including authorities and Jaibaná. The data were analyzed according to Constructivist Grounded Theory, using NVIVO software.

**Results.:**

The central category «Malaria: a disease that accompanies indigenous communities» emerged, supported by three subcategories: «Disease from the West,» which moves with people, can be prevented, and is always present; «The mosquito as the master of the disease,» which is controlled through training, cleanliness and hygiene, use of mosquito nets, and organic food; and «Health Care for Indigenous People with Malaria,» which begins with the Jaibaná, who controls the spirits that hinder the process and Western treatment.

**Conclusions.:**

Communities perceive malaria as a Western disease and attribute its origin and transmission to mosquitoes, without recognizing humans as the main source of outbreaks. The Jaibaná plays a fundamental role in patient care, addressing spiritual conditions that complement Western treatment. Although the incorporation of ancestral practices into health care for Indigenous communities is recommended, this is done empirically and based on public demand.

## INTRODUCTION

Malaria is a disease with a high morbidity and mortality rate, caused by protozoa of the genus *Plasmodium*, transmitted to humans through the bite of female *Anopheles* mosquitoes, by direct inoculation or by vertical transmission ^1^.

According to the 2023 annual malaria report from the World Health Organization (WHO), in 2022, 249 million cases were registered, and 608,000 deaths were estimated. Africa continues to account for 95% of cases and 96% of malaria deaths worldwide. WHO recognizes that, although significant progress has been made in the fight against this disease, it continues to be a major global public health problem ^2^.

In Colombia, up to epidemiological week 45 of 2024, there were 106,934 cumulative cases, of which 1875 were complicated, with the department of Chocó presenting the highest number of cases (35.4%). This disease mainly affects communities such as Afro-Colombians (30.3%) and Indigenous people (24%), and is concentrated, above all, in rural areas of the country ^3^.

Ancestral practices seek the eradication or treatment of diseases such as malaria through the use of different parts of plants considered medicinal, prepared as infusions, syrups, poultices, or powders ^4^. Griffith et al. documented several practices carried out by the «inadule» or traditional physician, among which the «pipe smoking,» the «cacao burning,» and «chili pepper» stand out ^5^. Pereira et al. found that 36% of respondents trust non-medical personnel, such as «the traditional healer,» who is capable of resolving the pathology using traditional methods instead of conventional ones ^6^.

In Colombia, some Indigenous communities treat malaria through the use of medicinal plant drinks and baths mixed with medications, liquor, or fruits ^7^. However, these practices sometimes fail to cure or improve the disease, and even then, communities sometimes avoid seeking treatment in health institutions ^8^. It should be noted that Indigenous communities continue to employ treatments and practices that are adequate for them, but which can lead to different complications and even death ^7^. Molineros-Gallón states that the community is aware of the disease, but the various practices they employ do not protect them against it ^8^.

Despite the efforts of health entities to prevent malaria, community actions do not seem to be consistent with the knowledge transmitted, as the number of cases remains high ^9^. A study on therapeutic adherence in malaria identified poverty, the use of traditional medicine, and deficiencies in health centers as significant barriers ^10^. However, some communities tend to link therapeutic treatment for malaria not only with biomedical models, but also with sociocultural and popular perceptions and representations. This favors a comprehensive understanding of the social representations and perceptions of malaria as well the qualitative improvements in control programs ^11^^,^^12^.

Therefore, the objective of the present study was to explore the way in which Indigenous communities coexist with malaria, through the recognition of the meanings they construct about the disease, its treatment, and care.

**KEY MESSAGES**

Motivation for conducting the study. The study was conducted due to the high incidence of malaria, despite existing prevention and control strategies. It was considered that the communities’ practices and beliefs influence malaria cases.

Main findings. Indigenous communities, although aware of the diagnosis and treatment of malaria, initially resort to traditional medicine to treat initial symptoms and differentiate it from other diseases.

Public health implications. It is crucial that health policies explicitly recognize and incorporate the role and scope of traditional medicine in the comprehensive management of malaria.

## MATERIALS AND METHODS

### Study design

A qualitative research approach was adopted, and through an ethnographic study, the way Indigenous communities experience and address malaria was explored. Through participant observation and interviews, the aim was to understand the meanings they give to their experiences, recognizing the «emic» and «ethic» perspectives ^13^.

### Participant selection

Six regions of the department of Chocó, Colombia, with a high prevalence of malaria, where Indigenous communities belonging to the Emberá Dóbida, Chamí, Katíos, and Éyabida ethnicities reside, were visited. The communities had an average of 633 inhabitants each, as described in Table 1. The visits were carried out as part of a medical mission to educate these communities about the care and treatment of malaria.

**Table 1:** Characteristics of the Communities Visited.

Region	***Resguardo* (Indigenous Reserve)**	Communities	Indigenous Ethnicity	Approx. No. of Inhabitants	Date of Visit	Hours of Observation
Playa Alta, Municipality of Quibdó	El 20, 90 and Playa Alta	Playa Alta, El 20, and El 90	Emberá Dóbida, Chamí, and Katíos	700	October 2022	40
Alto Atrato Zone, Municipality of Lloró	Tegavera	Tegavera, Peña India, Hurtado, and Platino	Emberá Dóbida	800	February 2023	40
Municipality of Unguía	Tanela	Ziparado, Tumburrula, and Citara	Emberá Dóbida and Éyabida	650	April 2023	40
Municipality of Quibdó	El 21	El 21 and Guachosa	Emberá Dóbida	400	June 2023	40
Municipality of Nuquí	Alto Rio Pangui	Yucal and Guadual	Emberá Dóbida	500	August 2023	40
Municipality Quibdó Nécora	Paratudo	Pueblo Nuevo Icho, Nécora, Baratudo, Peñalisa, and El Tigre	Emberá Dóbida	750	November 2023	40

Source: Authors’ own creation.

Between October 2022 and November 2023, a research team carried out participant observation under the participant-as-observer modality, dedicating an average of four days per visit to each community. During this time, the team not only participated in educational and medical assistance activities but also conducted a geographical reconnaissance, visited homes, lived with some families, supported the organization of spaces, attended community meetings, and spoke with representatives of the community.

The role of health providers favored the establishment of rapport during access and interaction, which allowed them to share their routines, dynamics, and daily practices. Likewise, it allowed them to maintain a critical and analytical distance.

In addition, through convenience sampling ^14^, semi-structured interviews were conducted with 25 people, including leaders, governors, health promoters, patients, teachers, Jaibaná, mothers, a traditional midwife, and a manager of an Institución Prestadora de Servicios de Salud (IPS) (Health Service Provider Institution).

### Data collection

The data collection team was made up of a physician-anthropologist who guided six medical students. Before fieldwork, the team participated in two preparation workshops on participant observation and interview techniques. Scripts for both activities were prepared, which are described in Table 2.

**Table 2:** Observation and Interview Guides.

Information Collection Strategy	Central Question or Topic	Secondary Questions or Topics
Participant Observation	Community Recognition	- Mapping and general and specific normative systems. - Malaria cases in the community. - Structures or places of risk for malaria transmission. - Community context.
	Social Dynamics	Location of health centers and observe: - Actions, language, and attitudes of the Indigenous people and the health personnel. - Communication, interaction, and relationship process.
		Identify Jaibanás, traditional healers, among others, and observe: - Dynamics of people when they go to a Jaibaná, traditional healer, among others. - Actions, language, and attitudes of the Jaibaná, traditional healer, among others. - Communication, interaction, and relationship process between the Jaibaná, traditional healer, or others, and the Indigenous people. - Gather the recommendations and indications given during the consultation.
	Personal Dynamics	- Describe what a person does when they have malaria. - Describe the daily life of the Indigenous person with malaria. - Describe how they seek medical attention. - Observe adherence and application of treatment.
	Family Dynamics	- Family dynamics. - Roles of family members. - Practices, actions, customs, among others, that families carry out inside the home regarding the management, care, and treatment of malaria.
Semi-structured Interview	How is the care process for an Indigenous patient with suspected malaria?	- How is the malaria diagnosis process? - What differences are there in the care of the Indigenous patient compared to other patients? -How is communication established with the Indigenous patient during care? - What beliefs, prejudices, prior knowledge, among others, do Indigenous patients usually express about malaria?
	How is the treatment for malaria in Indigenous patients?	- What treatment is given to an Indigenous patient with malaria? - What recommendations are given to patients with malaria? - What are the main goals or objectives of malaria treatment? - How is treatment adherence monitored?
	How does the Indigenous community participate in the care and management of patients with malaria?	- What are the support networks for Indigenous patients when they become ill with malaria? - What is the relationship or participation of representative community agents such as Jaibanás, traditional healers, among others? - What alternative therapies do you know that Indigenous patients usually perform to support malaria treatment? - What practices of the Indigenous communities surrounding malaria management do you perceive favor or hinder patient treatment?

Fuente

The functions were distributed among the members, covering the observation of the community’s macrocontext and its social, personal, and family dynamics, as well as conducting the interviews. At the end of each day, meetings were held to review and triangulate the observers’ perspectives.

For field access, contact was established with the chief counselor of the Association of Indigenous Traditional Authorities of the Emberá Dóbida, Katío, Chamí, and Dule Peoples (ASOREWA), who authorized the entry and performance of the study. Then, a formal request and a copy of the project were sent to the authorities of each community.

With the authorization of the Indigenous governors, the field was entered, and a non-intrusive immersion was carried out, including an induction and an initial reconnaissance of the community. Key informants were contacted, such as botanists, traditional physicians or Jaibaná, teachers, and health promoters.

Observation records were made in field diaries, and interviews were recorded on audio devices; five of them were conducted with the support of a translator. The information from the field diary and the interview recordings was transcribed textually in a word processor, stored, and coded.

Both the duration of stay in the field and the number of interviews were determined by sampling and theoretical saturation. That is, after the emergence of the categories during the analysis, participants were selected, and situations and practices of the communities were sought that contributed to their theoretical development. As soon as data saturation for each category was reached, data collection was considered complete.

### Analysis and findings

The analysis was based on Charmaz’s proposal on constructivist grounded theory ^15^. It was developed as an iterative process, constantly moving between the emic and ethic perspectives. It began with open coding that allowed the emergence of an initial structure of categories and subcategories that delimited patterns and typified behaviors, experiences, and meanings, among others. In focused coding, the categories were related and compared with their respective subcategories and units or concepts that constituted them.

Data collection and analysis were carried out concurrently. This contributed to the saturation of the categories. Data analysis was supported by NVIVO 1.6 software.

### Ethical and rigor aspects

The project was presented and endorsed by the Ethics Committee for Health Research of the Pontificia Bolivariana University of Medellín, through Act N° 11 of 2022. Likewise, the report adhered to the criteria of the Consolidated criteria for reporting qualitative research (COREQ) guide (16). In addition, the rigor criteria of qualitative research of credibility, auditability, and transferability were taken into account, which are described in Table 3.

**Table 3:** Rigor Criteria of the Study.

Criterion	Implemented Actions
Credibility	Reflexivity exercises were carried out through recordings in field diaries of the prejudices, preconceptions, stereotypes, among other aspects, that were held or emerged during the process. These were shared and discussed in periodic meetings of the research team.
	Preparation workshops for information collection were held prior to the fieldwork.
	The researchers were in charge of information collection, transcription, and analysis.
	Information was recorded on audio devices and in field diaries.
	Triangulation of information collection techniques was performed using interview and participant observation.
	Investigator triangulation was performed in both the fieldwork and the analysis process.
	Information collection was guided by theoretical sampling.
Auditability	The research was shared with academic communities and peer reviewers.
Transferability	The context and the conditions and characteristics of the regions and participants were described.

Fuente

## RESULTS

Twenty-five inhabitants from various Indigenous communities participated, with 68% of them being men. Among the participants were Indigenous governors (24%), health promoters (20%), community leaders (12%), and patients (12%). The participation of teachers, Jaibaná, mothers, a traditional midwife, and a manager of an Indigenous IPS was also included. Other sociodemographic characteristics are detailed in Table 4.

**Table 4:** Sociodemographic Characteristics of the Indigenous People Interviewed (n=25).

Variable		n	%
Sex			
	Male	17	68.0
	Female	8	32.0
Community			
	Yucal	5	20.0
	El Veintiuno	5	20.0
	Playa Bonita	4	16.0
	El Noventa	4	16.0
	Hurtado	4	16.0
	Peña India	3	12.0
Role			
	Governor	6	24.0
	Health Promoter	5	20.0
	Leader	3	12.0
	Patient	3	12.0
	Teacher	2	8.0
	Jaibaná	2	8.0
	Mother	2	8.0
	Traditional Midwife	1	4.0
	Indigenous IPS Manager	1	4.0

Source: Sociodemographic data of the participants

IPS: Health Service Provider Institution

**Figure 1:**
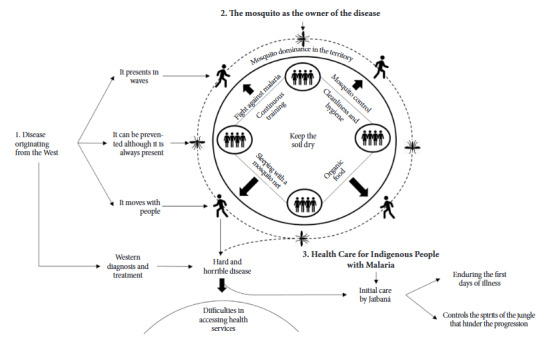
Graphical representation. Malaria, a disease that accompanies the Indigenous community

Source: Structure of analysis

From the data analysis, a central category emerges called «Malaria: a disease that accompanies Indigenous communities,» supported by three subcategories: «Disease from the West,» «The mosquito as the master of the disease,» and «Health Care for Indigenous People with Malaria.» Figure 1 describes the structure of the subcategories, relating them to each other to generate a theoretical schema of the studied phenomenon.

### Disease from the West

Malaria, or paludismo as many Indigenous people called it, is considered a disease that came from the West. Malaria has always been present in the community and has been perceived as a «hard and horrible» disease that can lead to death. However, they believe it can be prevented and cured, although not through the traditional healer or herbalist, which is why «The patient doesn’t have to be here if the Western physician is the one who should treat him» (DC01).

Malaria was conceived as a disease of «water wells» that is contracted, mainly, after five in the afternoon. Being on the banks of the river, in wells, or fish hatcheries, was considered a risk for contracting the disease, as these are places where the mosquitoes that affected people lived, as the following participant described: «Malaria is produced from a well or a ditch, or a little container... it is produced in the water and after it comes out of the water it bites one, in the early morning or in the afternoon, after five in the afternoon» (DC01).

Malaria occurred in waves, as there were times when cases increased, affecting several people at the same time. Likewise, the inhabitants considered that the disease moved with people, as they observed that when someone traveled to other areas and acquired malaria, upon returning, the family and other people in the community became ill. This was described in the following account: «The person only serves as a means to expand malaria... and if there is a mosquito here, of course it is possible that they transmit it» (DC05).

### The mosquito as the master of the disease

The Indigenous people were clear that the transmission of the disease occurred through the bite of the mosquito, which has dominance over the territory, and some believed that it is born with the disease. In this regard, the account of the disease always begins with the mosquito or zancudo, as if the disease were its own. The Indigenous people recognized that it is the mosquito that transmits it from person to person, as the following participant expressed: «Everything [is] related expressly to the mosquito, which is always there, with whom they share the territory, which gets agitated at certain times of the year» (DC02).

Malaria prevention consisted of an unending struggle to control the mosquito and was based on four strategies: 1) cleanliness, hygiene, and keeping the land dry; 2) sleeping with a mosquito net; 3) eating natural products; and 4) the presence of someone who knows about the disease.

Regarding the cleanliness and hygiene of the territory and homes, they learned to classify garbage and eliminate everything that favors mosquito reproduction. This was observed in the following situation: «Every day we have to keep the community clean, we have to dry the puddle, we have to drain where that mud pit is, where the ground is soft, we have to drain so that it hardens and doesn’t collect water» (DC05).

Sleeping with a mosquito net prevents mosquitoes from biting people. Its effectiveness was based on it remaining clean and impregnated with substances that acted as repellents, as one resident mentioned: «It’s about taking good care of oneself and sleeping under a mosquito net [and] impregnating the mosquito net so that the mosquitoes that arrive... all those that arrive to stick to the mosquito net at night then die» (DC05).

In relation to diet, they associated that plants and natural or organic foods were a source of strength and gave the body resistance to the disease, as the following participants indicated: «My mom told me that before, there was hardly any disease because they consumed natural things, that is, natural plants» (DC05).

The presence of health promoters or people who knew about malaria allowed for training and education of the community on transmission mechanisms, garbage management, water treatment, and use of the mosquito net, as described below: «With this workshop they are giving us, I understand that there are bacteria or diseases that one can get more complicated than one thought. The most important thing is to be aware and vigilant of which disease is falling and to analyze and study which remedy can also work» (DC05). «Here they explain to us in the training that a mosquito transmits it» (DC 04).

### Health Care for Indigenous People with Malaria

For the management of malaria, considered a Western disease, one had to go to the hospital, despite the Indigenous knowledge about the management of ailments and diseases, as the following quote reflected: «If they don’t have a natural disease that affects us, then yes, they can take them to the hospital so that, based on their function as a physician, they can be saved» (DC05). However, since access to Western medicine was neither easy nor timely, they usually stayed in the community for the first few days, a period during which the Jaibaná or the herbalist physician provided initial care.

The Jaibaná had the capacity to monitor the Western disease and the disease that affected the spirit. He knew that he could not cure malaria; however, he could manage the initial symptoms, especially fever. Likewise, he had the ability to speak with the spirits, who told him whether the disease was from the West or from them, and if the person would recover. As the following participant related: «When a child or adult gets sick, you have to stay up all night with the patients, and then through the spirit, they tell which patients can be attended to» (DC01). The Jaibaná’s intervention facilitated the care pathway for the person with malaria, as he acted by performing a **«triage»** that allowed for determining whether the person should be transferred to a hospital.

The Jaibaná made empirical use of plants that, although they did not cure the disease, did cushion it and supported the care of the sick person. This was based on the belief that there are «drinks that lift the sick person,» as the following Jaibaná related: «He does his treatment, that is... his rituals, he puts aguardiente (a type of liquor) in a little glass, they place it there in front, with the power of his staff, because his staff always has his power, only in the staff [... ] because we have that communication with nature, as well as with the animals. They call, they talk, so they ask them what disease they have» (DC05).

There were situations where the sick person was affected by **«spirits of the jungle»** or presented afflictions such as the **«evil eye»**. This could hinder the diagnosis of the disease and could obstruct or complicate the person’s evolution. These ailments were cured by the Jaibaná so that the Western treatment could be effective. This was observed in the following account: «In this patient, it seemed to be related to a spiritual disease that did not allow them to overcome malaria; a simple ceremony, with prayers and energies, was missing so that the patient would recover, and indeed, she got better, at least clinically» (DC06). Thus, in the community, there were two forms of salvation, the Jaibaná and the Western physician, which were considered complementary.

Some communities perceived an abandonment by the health system because the health centers or hospitals were distant from the territories where they lived, as the following Indigenous person related: «It has been more than three years since any health commission arrived in the community» (DC02), «Not everyone has the opportunity to go there to get tested and receive the pharmacological treatment at the health center, which is why they decide to supply themselves with Aralen (Chloroquine), an effective Western treatment against malaria, which they buy freely in pharmacies» (DC06). This implied that the sick person had to leave the community, travel long paths, with variable and difficult geographies to traverse. Likewise, traveling to the hospital was expensive for them, so sometimes, if the health condition was not serious, they preferred to stay in the community.

## DISCUSSION

Our findings indicate that Indigenous communities accept malaria as a foreign disease, adopting Western treatments and recognizing the mosquito as a vector. Despite this, geographical and economic barriers to health centers often prevent them from seeking care. In this context, the figure of the Jaibaná is crucial, as he manages the initial symptoms, determines the need for external care, and treats the spiritual ailments that could affect the efficacy of Western treatment.

Malaria is recognized as a foreign disease that was introduced into Indigenous communities, never to leave them. It is related to migration and the movement of infected people through their territories. Long *et al*. propose considering travelers from endemic areas with cyclic, paroxysmal symptoms and unexplained fever as a malaria case ^17^.

Our results confirm that the inhabitants of the communities perceived that when someone acquired malaria in another area, upon returning, the family and other people in the community became ill. Between 2017 and 2021, imported cases from border countries increased in China ^18^. The foregoing highlights the need to strengthen the collaboration and cooperation of local and national authorities to improve malaria surveillance and response ^18^^,^^19^.

Malaria is associated with eco-epidemiological factors and the presence of susceptible humans in rural and wooded areas ^20^. It is proposed to consider seasonal, temporal, and spatial variations, as well as climatic and environmental factors, to optimize the management and treatment of the disease ^21^^,^^22^. This, taking into account that, as observed in our study, the communities already have a basic learning of care and environment management to prevent the disease.

Mosquito control is the main way to prevent malaria ^23^. Attacking parasites in mosquitoes helps model epidemiological frameworks, control drug resistance, and prolong the useful life and clinical benefit of antimalarials ^24^^,^^25^. Biological control, using natural enemies of pests, can overcome the negative effects of using chemical insecticides ^26^.

Aquatic habitats influence the transmission and persistence of the disease; their elimination contributes to limiting malaria transmission ^27^, just as the Indigenous communities that participated in the research do. Dakorah et al. propose distributing insecticide-treated nets, performing residual spraying, and applying mass prophylaxis for effective control ^28^.

In the health care for Indigenous people with malaria, the Jaibaná provides initial care and directs the treatment. In this regard, the importance of traditional healers in the treatment of malaria is highlighted, suggesting initiatives related to knowledge transmission, conservation of medicinal plants, and the integration of health care ^29^.

Ethnomedical and ethnobotanical knowledge are important in primary health care in Indigenous communities, generating more confidence than modern medicine ^30^^-^^32^. It is recognized that, to treat malaria, there are communities that use both phytotherapy and pharmaceutical drugs, showing pragmatic medical pluralism ^33^.

It was identified that Indigenous communities combine conventional medical care with elements of their medical tradition. The cure of the disease is associated with the compatibility of the drug with the blessing of God ^34^. This is related to our findings, since the Jaibaná communicates with the spirits of nature to guide the management of the sick person.

The integration of local knowledge in malaria control is important. In this sense, Indigenous communities believe they can positively modify their environment to reduce malaria ^35^. Thus, malaria control policies must support practices and knowledge, preserving the historical context of Indigenous communities and protecting current professionals and practices ^36^.

A limitation of public health is the lack of medical attention for populations infected with malaria who live in hard-to-reach areas ^20^. Our findings show that, although communities know they should seek medical attention, the lack of resources, transportation, and remoteness of health centers hinder timely access.

Regarding study limitations, the language of some communities and the researchers’ lack of knowledge of it limited the fluidity of certain conversations, since they required the mediation of a translator. Furthermore, the lack of knowledge of Indigenous customs and the inexperience of some members of the research team in data collection through participant observation required more time to build trust during fieldwork.

It is concluded that Indigenous communities consider malaria a Western disease, which is always present, manifests in waves, and moves with people. They attribute its origin and transmission exclusively to mosquitoes, without identifying the human being as the main source of outbreaks. Efforts to eradicate malaria are perceived as an unending struggle to control the mosquito.

Communities recognize that malaria requires Western management; however, geographic and health service coverage barriers were identified that limit timely diagnosis and treatment. The Jaibaná is the figure who, within the communities, maintains and cares for the sick person, and helps them resolve spiritual ailments so that Western treatment can be effective.

Despite the fact that scientific literature and health policies recommend the incorporation of ancestral practices in the health care of Indigenous communities, the Jaibaná’s intervention is carried out empirically and based on public demand, since the guidelines and protocols for the management of malaria do not include their participation.
