# Supplementary material for: Between their own and the western: the experience of Emberá communities in Chocó with Malaria
Source: Rev Peru Med Exp Salud Publica. 2025 Nov 24;42(4):361–9. doi: 10.17843/rpmesp.2025.424.14846 (PMC12879987; doi:10.17843/rpmesp.2025.424.14846)
Supplement: Translation

# Translation: Entre lo propio y lo occidental: la experiencia de comunidades Emberá del Chocó con la Malaria

**Authors:** Camilo Duque-Ortiz, Stefany Osorno-Sánchez, Suatny Dayana Gutierrez-Asprilla, Maria Jose Garcia-Tirado, Juliana Nieto-Betancurt, Luisa Fernanda Guzman-Sánchez, Sara Montoya-Cedula, Marjorie Pérez-Villa, Luz Elena Botero-Palacio, Cristian Vera-Marin, José Mauricio Hernández-Sarmiento

**Affiliations:** 1 Universidad Pontificia Bolivariana, Medellín, Colombia.; 2 Grupo de Investigación Cuerpos y Contextos, Laboratorio De Osteología Antropológica y Forense (LOAF) del Departamento de Antropología, Universidad de Antioquia, Medellín, Colombia.

**Keywords:** Malaria, Mosquitos Vectores, Población Indígena, Medicina Tradicional, Conducta Social

## Abstract

**Objetivo.:**

Explorar cómo las comunidades Emberá del Chocó, Colombia, conviven con la malaria, a través del reconocimiento de los significados que construyen sobre la enfermedad, su tratamiento y cuidado.

**Materiales y métodos.:**

Estudio con enfoque etnográfico. Entre octubre de 2022 y noviembre de 2023, se llevaron a cabo 250 horas de observación participante en seis comunidades indígenas Emberá; y 25 entrevistas a miembros de las comunidades, incluyendo autoridades y jaibanás. Los datos se analizaron según la propuesta de la Teoría Fundamentada Constructivista, utilizando el software NVIVO.

**Resultados.:**

Emerge la categoría central «La malaria: enfermedad que acompaña a las comunidades indígenas», sustentada en tres subcategorías: «enfermedad proveniente de occidente» que se mueve con las personas, se puede prevenir y siempre está presente; «el mosquito como dueño de la enfermedad», el cual se controla mediante capacitación, limpieza y aseo, uso de toldillo y alimentación orgánica; y « la atención en salud del indígena con malaria» que inicia con el jaibaná quien controla a los espíritus que entorpecen la evolución y el tratamiento occidental.

**Conclusiones.:**

Las comunidades perciben la malaria como una enfermedad occidental y atribuyen su origen y transmisión a los mosquitos, sin reconocer al ser humano como la principal fuente de brotes. El jaibaná asume un rol fundamental en el cuidado del enfermo, atendiendo afecciones espirituales que complementan el tratamiento occidental. Aunque se recomienda la incorporación de prácticas ancestrales en la atención en salud de las comunidades indígenas, esta se realiza de forma empírica y por demanda de la población.

## INTRODUCCIÓN

La malaria es una enfermedad con alta tasa de morbilidad y mortalidad, causada por protozoarios del género *Plasmodium*, transmitidos al ser humano a través de la picadura de mosquitos hembra del género *Anopheles*, por inoculación directa o por transmisión vertical ^1^.

Según el informe anual sobre la malaria del 2023 de la Organización Mundial de la Salud (OMS), en 2022, se registraron 249 millones de casos y se estimaron 608 000 de muertes. África continúa representando el 95% de los casos y el 96% de las muertes por malaria a nivel mundial. La OMS reconoce que, si bien se han logrado avances significativos en la lucha contra esta enfermedad, aún sigue siendo un importante problema de salud pública global ^2^.

En Colombia, hasta la semana epidemiológica 45 de 2024 se contabilizaban 106 934 casos acumulados, de los cuales 1875 fueron complicadas, siendo el departamento del Chocó el que presentó el mayor nfúmero de casos (35,4%). Esta enfermedad afecta principalmente a comunidades como los afrocolombianos (30,3%) y los indígenas (24%), y se concentra, sobre todo, en áreas rurales del país ^3^.

Las prácticas ancestrales buscan la erradicación o el tratamiento de enfermedades como la malaria mediante el uso de diferentes partes de plantas consideradas medicinales, preparadas como infusiones, jarabes, emplastos o polvos ^4^. Griffith et al., documentaron varias prácticas realizadas por el «inadule» o médico tradicional, entre las que destacan la «fuma de la pipa», la «quema del cacao» y de «ají» ^5^. Pereira et al. encontraron que un 36% de los encuestados confían en personal no médico, como «el curandero», capaz de resolver la patología utilizando métodos tradicionales en lugar de los convencionales ^6^.

En Colombia, algunas comunidades indígenas tratan la malaria mediante el uso de bebidas y baños con plantas medicinales mezcladas con medicamentos, licor o frutos.^7^ No obstante, estas prácticas en ocasiones no logran curar o mejorar la enfermedad y, aun así, las comunidades a veces evitan ser tratadas en instituciones de salud ^8^.

Cabe destacar que las comunidades indígenas siguen empleando tratamientos y prácticas que para ellos son adecuados, pero que pueden llevar a diferentes complicaciones e incluso la muerte ^7^. Molineros-Gallón afirman que la comunidad conoce la enfermedad, pero las diferentes prácticas que realizan no los protegen contra esta ^8^.

A pesar de los esfuerzos de las entidades de salud para prevenir la malaria, las acciones comunitarias no parecen ser coherentes con el conocimiento transmitido, ya que el número de casos sigue siendo elevado ^9^. Un estudio sobre la adherencia terapéutica en la malaria identificó la pobreza, el uso de la medicina tradicional y las deficiencias en los centros de salud como barreras significativas ^10^. Sin embargo, algunas comunidades tienden a vincular el tratamiento terapéutico para la malaria no solo con modelos biomédicos, sino también con percepciones y representaciones socioculturales y populares. Esto favorece una comprensión integral de las representaciones y percepciones sociales de la malaria y mejoras cualitativas de los programas de control ^11^^,^^12^.

Por lo tanto, el objetivo del presente estudio fue explorar la manera como las comunidades indígenas conviven con la malaria, a través del reconocimiento de los significados que construyen sobre la enfermedad, su tratamiento y cuidado.

**MENSAJE CLAVE**

Motivación para realizar el estudio. El estudio se realizó debido a la alta incidencia de malaria, a pesar de existir estrategias de prevención y control. Se consideró que las prácticas y creencias de las comunidades influyen en los casos de malaria.

Principales hallazgos. Las comunidades indígenas, aunque conocen el diagnóstico y tratamiento de la malaria, inicialmente recurren a la medicina tradicional para tratar los síntomas iniciales y diferenciarla de otras enfermedades.

Implicancias en salud pública. Es crucial que las políticas de salud reconozcan e incorporen explícitamente el papel y alcance de la medicina tradicional en el manejo integral de la malaria.

## MATERIALES Y MÉTODOS

### Diseño del estudio

Se adoptó un enfoque cualitativo de investigación y mediante un estudio etnográfico que se exploró la forma en que las comunidades indígenas vivencian y abordan la malaria. A través de observación participante y entrevistas, se buscó comprender los significados que otorgan a sus experiencias, reconociendo las perspectivas «emic» y «ethic» ^13^.

### Selección de participantes

Se visitaron seis regiones del departamento de Choco, Colombia, con alta prevalencia de malaria, donde se encuentran asentadas comunidades indígenas pertenecientes a las etnias Emberá Dóbida, Chamí, Katíos y *Éyabida*, con un promedio de 633 habitantes por comunidad, como se describe en la tabla 1. Las visitas se realizaron como parte de una misión médica para educar a estas comunidades sobre el cuidado y tratamiento de la malaria.

**Tabla 1:** Características de las comunidades visitadas.

Región	Resguardo	Comunidades	Etnia indígena	N° aproximado de habitantes	Fecha de la visita	Horas de observación
Playa alta, Municipio de Quibd**ó**	El 20, 90 y Playa Alta	Playa Alta, el 20 y el 90	Emberá Dóbida, Chamí y Katíos	700	Octubre de 2022	40
Zona de Alto Atrato, Municipio de Lloró	Tegavera	Tegavera, Peña India, Hurtado y Platino	Emberá Dóbida	800	Febrero de 2023	40
Municipio de Unguía	Tanela	Ziparado, Tumburrula y Citara	Emberá Dóbida y Eyábida	650	Abril de 2023	40
Municipio de Quibdó	El 21	El 21 y Guachosa	Emberá Dóbida	400	Junio de 2023	40
Municipio de Nuquí	Alto Rio Pangui	Yucal y Guadual	Emberá Dóbida	500	Agosto de 2023	40
Municipio Quibdó Nécora	Paratudo	Pueblo Nuevo Icho, Nécora, Baratudo y Peñalisa y el Tigre	Emberá Dóbida	750	Noviembre de 2023	40

Fuente: Elaboración propia.

Entre octubre de 2022 y noviembre de 2023, un equipo de investigación llevó a cabo una observación participante bajo la modalidad de participante como observador, dedicando un promedio de cuatro días por visita a cada comunidad. Durante este tiempo, el equipo no solo participó en las actividades educativas y de asistencia médica, sino que también realizó un reconocimiento geográfico, visitó viviendas, convivió con algunas familias, apoyó en la organización de espacios, asistió a reuniones comunitarias y conversó con personas representativas de la comunidad.

El rol de proveedores de salud favoreció el establecimiento de rapport durante el acceso y la interacción, lo que permitió compartir sus rutinas, dinámicas y prácticas cotidianas. Así mismo, permitió mantener una distancia crítica y analítica.

Además, mediante un muestreo por conveniencia ^14^, se realizaron entrevistas semiestructuradas a 25 personas incluyendo líderes, gobernadores, promotores de salud, pacientes, profesoras, jaibanás, madres de familia, una partera y un gerente de una Institución Prestadora de Servicios de Salud (IPS).

### Recolección de datos

El equipo de recolección de datos estuvo conformado por un médico- antropólogo que guió a seis estudiantes de medicina. Antes del trabajo de campo, el equipo participó de dos talleres de preparación sobre observación participante y técnicas de entrevistas. Se elaboraron guiones para ambas actividades, los cuales se describen en la tabla 2.

**Tabla 2:** Guiones de observación y entrevista.

Estrategia de recolección de información	Pregunta o tópico central	Preguntas o tópicos secundarios
Observación participante	Reconocimiento de la comunidad	- Mapeo y sistemas normativos generales y específicos. - Casos de malaria en la comunidad. - Estructuras o lugares de riesgo para la transmisión de malaria - Contexto de la comunidad
	Dinámicas sociales	Ubicación de los centros de salud y observar: - Acciones, lenguaje y actitudes de los indígenas y el personal de salud - Proceso de comunicación, interacción y relación.
		Reconocer jaibanás, curanderos, entre otros y observar: - Dinámicas de las personas cuando acuden donde un jaibaná, curandero, entre otro - Acciones, lenguaje y actitudes del jaibaná, curandero, entre otro. - Proceso de comunicación, interacción y relación entre el jaibaná, curandero u otros y los indígenas. - Recopilar las recomendaciones e indicaciones que se dan en la consulta.
	Dinámicas personales	- Describir lo que hace una persona cuando tiene malaria. - Describir la cotidianidad del indígena con malaria. - Describir cómo buscan atención médica. - Observar adherencia y aplicación de tratamiento.
	Dinámicas familiares	- Dinámicas familiares. - Roles de los integrantes de la familia. - Prácticas, acciones, costumbres, entre otros, que realizan las familias en el interior del hogar en cuando al manejo, cuidado y tratamiento de la malaria.
Entrevista semiestructurada	¿Cómo es el proceso de atención de un paciente indígena con sospecha de malaria?	- ¿Cómo es el proceso del diagnóstico de la malaria? - ¿Qué diferencias hay en la atención del paciente indígena respecto a otros pacientes? - ¿Cómo se establece la comunicación con el paciente indígena durante la atención? - ¿Cuáles son las creencias, prejuicios, conocimientos previos, entre otros, que suelen manifestar los pacientes indígenas sobre la malaria?
	¿Cómo es el tratamiento de la malaria en paciente indígenas?	- ¿Cuál es el tratamiento que se le da a un paciente indígena con malaria? - ¿Cuáles son las recomendaciones que les dan a los pacientes con malaria? - ¿Cuáles son las metas u objetivos principales del tratamiento de la malaria? - ¿Cómo se hace seguimiento a la adherencia al tratamiento?
	¿Cómo participa la comunidad indígena en la atención y cuidado de los pacientes con malaria?	- ¿Cuáles son las redes de apoyo de los pacientes indígenas cuando se enferman con malaria? - ¿Cómo es la relación o participación de agentes comunitarios representativos como jaibanás, curanderos, entre otros? - ¿Qué terapias alternativas conoce usted que suelen realizar los pacientes indígenas para apoyar el tratamiento de la malaria? - ¿Qué prácticas de las comunidades indígenas alrededor del manejo de la malaria percibe que favorecen o dificultan el tratamiento de los pacientes?

Fuente: Elaboración propia.

Las funciones se distribuyeron entre los integrantes, abarcando la observación del macrocontexto comunitario y de las dinámicas sociales, personales y familiares, así como la conducción de las entrevistas. Al finalizar cada jornada, se realizaron reuniones para revisar y triangular las perspectivas de los observadores.

Para el acceso al campo se estableció contacto con el consejero mayor de la Asociación de Cabildos, Autoridades Tradicionales Indígenas Emberá Dóbida, Katío, Chamí y Dule (ASOREWA), quien autorizó el ingreso y realización del estudio. Luego, se envió una solicitud formal y una copia del proyecto a las autoridades de cada comunidad.

Con autorización de los gobernadores indígenas, se ingresó al campo y se realizó una inmersión no intrusiva, incluyendo una inducción y un reconocimiento inicial de la comunidad. Se contactó a informantes claves como: botánicos, médicos tradicionales o jaibaná, profesoras y promotores de salud.

Los registros de la observación se realizaron en diarios de campo y las entrevistas fueron grabadas en dispositivos de audio; cinco de ellas se realizaron con el apoyo de un traductor. La información del diario de campo y las grabaciones de las entrevistas se transcribieron textualmente en un procesador de texto, se almacenaron y codificaron.

Tanto la permanencia en el campo como el número de entrevistas se determinaron por muestreo y saturación teórica. Es decir, tras la emergencia de las categorías durante el análisis, se seleccionaron participantes y se buscaron situaciones y prácticas de las comunidades que contribuyeran a su desarrolló teórico. En cuanto se alcanzó una saturación de datos de cada categoría se consideró por concluida la recolección de datos.

### Análisis y hallazgos

El análisis se basó en la propuesta de Charmaz sobre la teoría fundamentada constructivista ^15^. Se desarrolló como un proceso iterativo, transitando constantemente entre las perspectivas emic y ethic. Se inició con una codificación abierta que permitió la emergencia de una estructura inicial de categoría y subcategorías que delimitaron patrones y tipificaron comportamientos, experiencias, significados, entre otros. En la codificación focalizada las categorías se relacionaron y compararon con sus respectivas subcategorías y unidades o conceptos que las constituyeron.

La recolección y análisis de la información se realizaron de manera concurrente. Esto contribuyó a la saturación de las categorías. El análisis de los datos se apoyó en el software NVIVO 1.6.

### Aspectos éticos y de rigor

El proyecto fue presentado y avalado por el Comité de Ética de Investigación en Salud de la Universidad Pontificia Bolivariana de Medellín, mediante el acta N° 11 del 2022. De igual forma, el reporte se adhirió a los criterios de la guía *Consolidated criteria for reporting qualitative research* (COREQ) ^16^. Además, se tuvieron en cuenta los criterios de rigor de la investigación cualitativa de credibilidad, auditabilidad y transferibilidad, los cuales se describen en la tabla 3.

**Tabla 3:** Criterios de rigor del estudio.

Criterio	Acciones implementadas
Credibilidad	Se hizo ejercicios de reflexividad por medio de registros en diarios de campos de los prejuicios, preconcepciones, estereotipos, entre otros aspectos, que se tenían o surgieron durante el proceso. Se compartieron y discutieron en encuentros periódicos del equipo investigador.
	Se realizaron talleres de preparación para la recolección de información previos al trabajo de campo.
	Los investigadores estuvieron a cargo de la recolección de información, transcripción y análisis de la misma.
	Se registró la información en dispositivos de audio y diarios de campo.
	Se hizo triangulación de técnicas de recolección de información con la entrevista y la observación participante.
	Se realizó triangulación de investigadores tanto en el trabajo de campo como el proceso de análisis.
	Se orientó la recolección de información por muestreo teórico.
Auditabilidad	La investigación fue compartida con comunidades académicas y pares evaluadores.
Transferibilidad	Se realizó la descripción del contexto y de las condiciones y características de las regiones y de los participantes.

Fuente: Elaboración propia.

## RESULTADOS

Participaron 25 habitantes de diversas comunidades indígenas, de los cuales el 68% eran hombres. Entre los participantes se encontraban gobernadores indígenas (24%), promotores de salud (20%), líderes comunales (12%) y pacientes (12%). También se contó con la participación de profesoras, jaibanás, madres de familia, una partera y un gerente de una IPS indígena. Otras características sociodemográficas se detallan en la tabla 4.

**Tabla 4:** Características sociodemográficas de los indígenas entrevistados (n=25).

Variable		n	%
Sexo			
	Masculino	17	68,0
	Femenino	8	32,0
Comunidad			
	Yucal	5	20,0
	El Veintiuno	5	20,0
	Playa Bonita	4	16,0
	El Noventa	4	16,0
	Hurtado	4	16,0
	Peña India	3	12,0
Líder			
	Gobernador	6	24,0
	Promotor de Salud	5	20,0
	Líder	3	12,0
	Paciente	3	12,0
	Profesora	2	8,0
	Jaibaná	2	8,0
	Madre de Familia	2	8,0
	Partera	1	4,0
	Gerente IPS Indígena	1	4,0

Fuente: Datos sociodemográficos de los participantes

IPS: Institución Prestadora de Servicios de Salud

Del análisis de los datos, emerge una categoría central denominada «la malaria: enfermedad que acompaña a las comunidades indígenas», sustentada en tres subcategorías: «enfermedad proveniente de occidente», «el mosquito como dueño de la enfermedad» y «la atención en salud del indígena con malaria». En la figura 1 se describe la estructura de las subcategorías, relacionándolas entre ellas para generar un esquema teórico del fenómeno estudiado.

**Figura 1:** Representación gráfica. La malaria, enfermedad que acompaña a la comunidad indígena.

Fuente: Estructura de análisis

### Enfermedad proveniente de occidente

La malaria, o paludismo como muchos indígenas la denominaron, es considerada una enfermedad que provino del occidente. La malaria siempre ha estado presente en la comunidad y se ha percibido como una enfermedad «dura y horrible» que puede llevar a la muerte. Sin embargo, creen que se puede prevenir y curar, aunque no a través del médico tradicional o yerbatero, por ello «El paciente no tiene que estar aquí si el médico occidental es el que lo debe tratar» (DC01)*.*

La malaria la concibieron como una enfermedad de «pozos de agua» que se contrae, principalmente, después de las cinco de la tarde. Estar a orillas del río, en pozos o criaderos de pescado, se consideró un riesgo para contraer la enfermedad, pues son sitios en los que habitaban los zancudos que afectaban a las personas, como lo describió el siguiente participante: «La malaria se produce de un pozo o una zanja, o un tarrito… en el agua se produce y después de que salga del agua lo pica a uno, en la madrugada o en la tarde, después de las cinco de la tarde» (DC01).

La malaria se presentaba por oleadas, ya que había momentos donde los casos aumentaban, afectando a varias personas al mismo tiempo. Asimismo, los habitantes consideraban que la enfermedad se movía con las personas, pues observaban que cuando alguien se desplazaba a otras zonas y adquiría la malaria, al regresar, la familia y otras personas de la comunidad se enfermaban. Esto se describió en el siguiente relato «La persona sirve únicamente como de, como de expandir la malaria… y si aquí hay zancudo, claro que es posible que lo transmita» (DC05)*.*

### El mosquito como dueño de la enfermedad

Los indígenas tenían claro que la transmisión de la enfermedad se daba por la picadura del mosquito quien tiene un dominio en el territorio y algunos creían que este nace con la enfermedad. Al respecto, el relato de la enfermedad lo inician, como siempre, con el mosquito o zancudo, como si la enfermedad fuera propia de este. Los indígenas reconocían que es el mosquito quien la transmitía de persona a persona, como lo expresó el siguiente participante: «Todo [está] relacionado expresamente con el mosquito; que siempre está allí, con quien comparten el territorio, que se alborota en ciertos periodos del año*»* (DC02).

La prevención de la malaria consistía en una lucha interminable para controlar el mosquito y se fundamentó en cuatro estrategias: 1) la limpieza, el aseo y mantener la tierra seca; 2) dormir con toldillo; 3) una alimentación con productos naturales; y 4) la presencia de alguien que conozca sobre la enfermedad.

En cuanto a la limpieza y el aseo del territorio y de las viviendas, aprendieron a clasificar las basuras y eliminar todo aquello que propiciara la reproducción del zancudo. Esto se observó en la siguiente situación: *«*A diario hay que mantener la comunidad limpia, hay que secar la charca, hay que drenar dónde está ese barrial, donde está blanda la tierra, hay que drenar para que endurezca y no recoja agua» (DC05).

Dormir con toldillo evitaba que el zancudo pique a las personas. Su efectividad radicaba en que permaneciera limpio e impregnado con sustancias que actuaran como repelentes, como lo mencionó un residente: «Es cuidarse mucho y dormir en toldillo [e] impregnar el toldillo para que los zancudos que lleguen … todo el que llegue a pegarse en la noche al toldillo entonces se muera» (DC05).

En relación con la alimentación, asociaban que las plantas y los alimentos naturales u orgánicos eran fuente de fortaleza y le daban resistencia al cuerpo frente a la enfermedad, como lo indicaron los siguientes participantes: «Mi mamá me decía que antes casi no había la enfermedad porque ellos consumían las cosas naturales, o sea, las plantas naturales» (DC05).

La presencia de promotores de salud o personas que conocían de la malaria permitió la capacitación y educación de la comunidad sobre los mecanismos de transmisión, el manejo de las basuras, el tratamiento de aguas y el uso del toldillo, tal como lo describen a continuación: «Con ese taller que nos están dando, comprendo que hay bacterias o enfermedades que uno se puede complicar más de lo que pensaba. Lo más importante es estar al tanto y pendiente de que enfermedad se está cayendo y analizar y el estudio de qué remedio puede servir también» (DC05). «Acá nos explican en la capacitación que eso lo transmite un zancudo» (DC 04).

### La atención en salud del indígena con malaria

Para el manejo de la malaria, considerada como enfermedad occidental, se debía acudir al hospital, a pesar del conocimiento indígena sobre el manejo de los males y las enfermedades, como lo reflejó el siguiente relato: «Si no tiene enfermedad natural que a nosotros nos afecta ahí sí puede llevar al hospital para que desde su función como médico se pueda salvar» (DC05). Sin embargo, como el acceso a la medicina occidental no era fácil ni oportuna, solían permanecer los primeros días en la comunidad, periodo en que el Jaibaná o el médico yerbatero hacía una atención inicial.

El Jaibaná tenía la capacidad de estar pendiente de la enfermedad occidental y de la enfermedad que afectaba al espíritu. Sabía que no podía curar la malaria, sin embargo, sí podía manejar los primeros síntomas, especialmente la fiebre. Asimismo, tenía la habilidad para hablar con los espíritus, quienes le indicaban si la enfermedad era de occidente o de ellos, y si se iba a recuperar. Como lo relató el siguiente participante: «Cuando un niño o adulto se enferma, toca trasnochar con los pacientes y después por medio del espíritu, ellos cuentan a qué pacientes se pueden atender» (DC01). La intervención del jaibaná facilitaba la ruta de atención de la persona con malaria, pues actuaba haciendo un «triage» que permitía determinar si la persona debía ser trasladada a un hospital.

El jaibaná hacía uso empírico de plantas que, si bien no curaban la enfermedad, sí la amortiguaban y apoyaban el cuidado de la persona enferma. Esto se fundamentaba en la creencia de que hay «bebidas que levantan al enfermo», como lo relató el siguiente jaibaná: «El hace su tratamiento, o sea ... sus rituales, le pone aguardiente en un vasito, colocan allí enfrente, con su poder de bastón, porque en los bastones siempre hay poder de él, solamente en el bastón […] porque nosotros tenemos esa comunicación con la naturaleza, tanto con los animales. Llaman, hablan, entonces ellos le preguntan qué enfermedad tiene» (DC05).

Hubo situaciones donde la persona enferma era afectada por «espíritus de la selva» o presentaba afecciones como el «mal de ojo». Esto podía dificultar el diagnóstico de la enfermedad y podía entorpecer o complicar la evolución de la persona. Dichos males los curaba el jaibaná para que así, el tratamiento occidental, pudiera ser efectivo. Esto se observó en el siguiente relato: «En esta paciente pareciera que se relacionó con una enfermedad espiritual que no le permitía vencer a la malaria; faltaba una ceremonia sencilla, de rezos y energías para que la paciente se recuperara, y efectivamente se mejoró, al menos clínicamente» (DC06). Así, en la comunidad había dos salvaciones, el jaibaná y el médico de occidente, los cuales fueron considerados como complementarios.

Algunas comunidades percibían un abandono del sistema de salud debido a que los centros de salud u hospitales se encontraban distantes a los territorios donde habitaban, como la relató el siguiente indígena «Hace más de tres años no llegaba ninguna comisión de salud a la comunidad» (DC02), «No todos tienen la oportunidad de ir hasta allá para hacerse la prueba y recibir el tratamiento farmacológico en el centro de salud, por lo cual deciden abastecerse por su cuenta con el Aralen (Cloroquina), tratamiento occidental efectivo contra la malaria, el cual compran libremente en las farmacias» (DC06). Esto implicaba que el enfermo debía salir de la comunidad, recorrer caminos largos, con geografías variables y difíciles de transitar. Asimismo, desplazarse al hospital les resultaba costoso, por lo que, en ocasiones, si la afección de salud no era grave, preferían quedarse en la comunidad.

## DISCUSIÓN

Nuestros hallazgos indican que las comunidades indígenas aceptan la malaria como una enfermedad foránea, adoptando los tratamientos occidentales y reconociendo el mosquito como vector. A pesar de esto, las barreras geográficas y económicas a los centros de salud a menudo les impiden buscar atención. En este contexto, la figura del Jaibaná es crucial, ya que maneja los síntomas iniciales, determina la necesidad de atención externa y trata las dolencias espirituales que podrían afectar la eficacia del tratamiento occidental.

Se reconoce que la malaria es una enfermedad foránea que fue introducida en las comunidades indígenas para nunca dejarlas. Está relacionada con la migración y el movimiento de personas infectadas a través de sus territorios. Long *et al.* proponen considerar como caso de malaria a viajeros procedentes de zonas endémicas con síntomas cíclicos, paroxísticos y fiebre inexplicable ^17^.

Nuestros resultados confirman que los habitantes de las comunidades percibían que cuando alguien adquiría la malaria en otra zona, al regresar, la familia y otras personas de la comunidad se enfermaban. Entre el 2017 y 2021, en China aumentaron los casos importados desde países fronterizos ^18^. Lo anterior resalta la necesidad de fortalecer la colaboración y cooperación de autoridades, locales y nacionales, para mejorar la vigilancia y respuesta a la malaria ^18^^,^^19^.

La malaria se asocia a factores eco-epidemiológicos y a la presencia de humanos susceptibles en zonas rurales y boscosas ^20^. Se propone considerar variaciones estacionales, temporales y espaciales, así como factores climáticos y ambientales, para optimizar el manejo y tratamiento de la enfermedad ^21^^,^^22^. Esto, teniendo en cuenta que, como se observó en nuestro estudio, las comunidades ya cuentan con un aprendizaje básico de cuidado y manejo del entorno para prevenir la enfermedad.

El control de los mosquitos es la principal forma de prevenir la malaria ^23^. Atacar los parásitos en los mosquitos ayuda a modelar marcos epidemiológicos y a controlar la resistencia a medicamentos y prolongar la vida útil y el beneficio clínico de los antipalúdicos ^24^^,^^25^. El control biológico, usando enemigos naturales de las plagas, puede superar los efectos negativos del uso de insecticidas químicos ^26^.

Los hábitats acuáticos influyen en la transmisión y persistencia de la enfermedad, su eliminación contribuye a limitar la transmisión de la malaria ^27^, tal como lo hacen las comunidades indígenas que participaron de la investigación. Dakorah et al. proponen distribuir mosquiteros tratados con insecticidas, realizar fumigación residual y aplicar profilaxis masiva para un control efectivo ^28^.

En la atención en salud de la malaria en indígenas, el jaibaná brinda la atención inicial y direcciona el cuidado. Al respecto, se destaca la importancia de los curanderos tradicionales en el tratamiento de la malaria, sugiriendo iniciativas relacionadas con la transmisión de conocimientos, la conservación de plantas medicinales y la integración de la atención sanitaria ^29^.

El conocimiento etnomédico y etnobotánico son importantes en la atención primaria en salud de comunidades indígenas, generando más confianza que la medicina moderna ^30^^-^^32^. Se reconoce que, para tratar la malaria, hay comunidades que usan tanto la fitoterapia como los medicamentos farmacéuticos, mostrando un pluralismo médico pragmático ^33^.

Se identificó que las comunidades indígenas combinan la atención médica convencional con elementos de su tradición médica. La cura de la enfermedad se asocia a la compatibilidad del fármaco con la bendición de Dios ^34^^).^ Esto se relaciona con nuestros hallazgos, pues el jaibaná se comunica con los espíritus de la naturaleza para orientar el manejo del enfermo.

Es importante la integración de conocimientos locales en el control de la malaria. En este sentido, las comunidades indígenas creen que pueden modificar positivamente su entorno para reducir la malaria ^35^. Así, las políticas para el control de la malaria deben apoyar las prácticas y conocimientos, preservando el contexto histórico de las comunidades indígenas y protegiendo a profesionales y prácticas actuales ^36^.

Una limitación de la salud pública es la falta de atención médica de las poblaciones infectadas con malaria que viven en áreas de difícil acceso ^20^. Nuestros hallazgos muestran que, aunque las comunidades saben que deben buscar la atención médica, la falta de recursos, transporte y lejanía de los centros de salud, dificultan el acceso oportuno.

En cuanto a limitaciones del estudio, el idioma de algunas comunidades y su desconocimiento por los investigadores limitaron la fluidez de ciertas conversaciones, ya que requirieron de la medicación de un traductor. Además, el desconocimiento de las costumbres indígenas y la inexperiencia de algunos integrantes del equipo investigador en la recolección de datos mediante la observación participante requirió más tiempo para generar confianza durante el trabajo de campo.

Se concluye que las comunidades indígenas consideran la malaria como una enfermedad occidental, que está siempre presente, que se manifiesta por oleadas y se mueve con las personas. Atribuyen su origen y transmisión exclusivamente a los mosquitos, sin identificar al ser humano como la principal fuente de brotes. Los esfuerzos para erradicar la malaria se perciben como una lucha interminable para controlar el mosquito.

Las comunidades reconocen que la malaria requiere de un manejo occidental, sin embargo, se identificaron barreras geográficas y de cobertura de los servicios de salud que limitan el diagnóstico y tratamiento oportuno. El jaibaná es la figura que, dentro de las comunidades, mantiene y cuida a la persona enferma, y la ayuda a resolver afecciones o males espirituales para que el tratamiento occidental puede ser efectivo.

A pesar de que la literatura científica y las políticas en salud recomiendan la incorporación de prácticas ancestrales en la atención en salud de comunidades indígenas, la intervención del jaibaná se realiza de forma empírica y por demanda de la población, ya que las guías y protocolos para el manejo de la malaria no incluyen su participación.
